# Ictal Asystole in a Patient With DEE due to an *FGF12* Pathogenic Variant: A Reminder to Monitor Cardiac Function

**DOI:** 10.1111/cge.70018

**Published:** 2025-07-10

**Authors:** Johanne Piotrowski, Élise Schaefer, Michael Kuntz, Virginie Bois, Franz Schieffer, Laurence Jesel, Margaux Biehler, Anne De Saint Martin, Sarah Baer

**Affiliations:** ^1^ Clinical Genetics Unit Institut de Génétique Médicale D'alsace (IGMA) Strasbourg France; ^2^ Cardiologie Infantile, Strasbourg University Hospital Strasbourg France; ^3^ Department of Pediatrics Hôpitaux Civils de Colmar Colmar France; ^4^ Division of Cardiovascular Medicine Strasbourg University Hospital Strasbourg France; ^5^ Laboratories of Genetic Diagnosis Strasbourg University Hospitals Strasbourg France; ^6^ Department of Neuropediatrics CréER, ERN EpiCare, Strasbourg University Hospitals Strasbourg France; ^7^ Institute for Genetics and Molecular and Cellular Biology, University of Strasbourg, CNRS UMR7104, INSERM U1258 Illkirch France

**Keywords:** ECG, epileptic encephalopathy, *FGF12*, *FHF1*, ictal asystole

## Abstract

We report the first known case of a 9‐year‐old male with early‐onset epilepsy, syncope, and ictal asystole–requiring pacemaker implantation at the age of seven–associated with a pathogenic variant in *FGF12*.

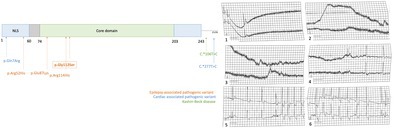

To the Editor,

*FGF12* (also known as FHF1 or Fibroblast Growth Factor 12) encodes a protein that binds to voltage‐gated sodium channels (NaV), playing a crucial role in enhancing neuronal excitability. Depending on the variant, it has been previously reported as implicated in developmental and epileptic encephalopathy 47 (DEE 47), cancer, pulmonary arterial hypertension, Kashin‐Beck disease, vestibular dysfunction, and Brugada syndrome [1].

A total of 27 patients with *FGF12* variants and severe DEE have been identified. To date, no specific cardiac rhythm disorders or ictal asystole have been described specifically in these individuals associated with epilepsy.

We present a 9‐year‐old male with early‐onset epilepsy, syncope, and ictal asystole requiring pacemaker implantation at the age of 7 years.

The patient was born at term with normal growth and no family history of epilepsy. His first seizure, a status epilepticus, occurred at 4 months and was treated with clonazepam and phenytoin. EEG, MRI, and metabolic tests were normal. Valproate was started at discharge.

Motor milestones were slightly delayed (walking at 22 months), but speech was normal. Despite treatment, monthly tonic–clonic seizures continued, often triggered by emotional stress. Seizures stopped between ages 2 and 4, allowing withdrawal of valproate. At age 4, a status epilepticus triggered by *Influenza A* required emergency treatment, and valproate was restarted.

At age 6, monthly seizures resumed, triggered by emotions or physical activity. These brief tonic seizures involved cyanosis but did not require emergency care. In addition, the patient had unexplained syncopal episodes. Cardiac echography revealed only a small atrial septal defect (6 mm, left‐to‐right shunt). A stress test was unremarkable. A holter ECG confirmed a 25‐s peri‐ictal asystole in an ambulatory record (Figure 1A) and a 10‐s asystole recorded during a tonic seizure in the hospital. Interictally, a probable vagally mediated sinus arrhythmia without significant pauses was noted. A single chamber pacemaker was implanted at the age of 7 years. At 3 years post‐pacemaker implantation, device activity was under 1%, indicating pacing was possibly limited to seizure‐related events.

**FIGURE 1:**
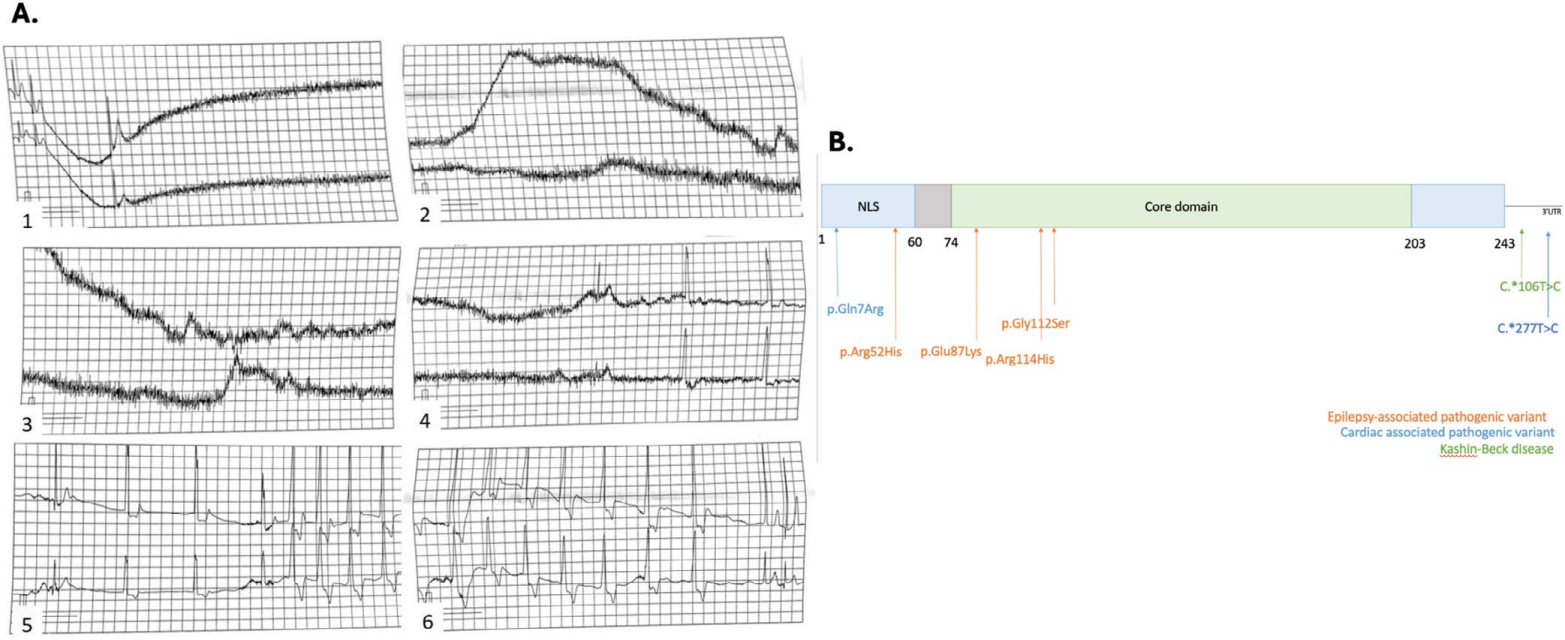
(A) Holter ECG at age 6 showing a 25‐s asystole during a seizure. Chronological excerpt shown. Calibration: 1 square = 0.2 s (horizontal), 0.5 mV (vertical). (B) FGF12 protein schematic (per Biadun et al.) with pathogenic variants: Orange (epilepsy), blue (cardiac disorders), green (Kashin‐Beck, 3′UTR 106T>C). (Transcript: NM_021032).

Seizures were controlled with Valproate and Zonisamide after Levetiracetam was discontinued from initial triple therapy.

Neuropsychological assessment at age 9 years revealed a normal but heterogeneous IQ, with mild difficulties in written language, cognition, and concentration. Difficulties in social interaction and atypical sensory processing were observed, although no formal diagnosis of autism was established. He attended mainstream education with the support of a special needs teaching assistant.

Trio whole exome sequencing (WES) was performed using the Illumina HiSeq 4000 platform, achieving 96% coverage of the exome at a depth of ≥ 10×. The WES analysis incorporated the following HPO terms: Seizure (HP:0001250), Autism (HP:0000717), and Sinus bradycardia (HP:0001688) and identified a *de novo* heterozygous *FGF12* [NM_021032: c.334G>A:p.(Gly112Ser)] variant. This recurrent pathogenic variant, which is not present in the gnomAD database, was reported as pathogenic in ClinVar. No other pathogenic variant has been found in the WES analysis.

We propose that the *FGF12* variant contributes to both epilepsy and ictal asystole in our patient, possibly via enhanced parasympathetic activation during seizures. *FGF12* belongs to the family of fibroblast growth factor homologues and is highly expressed in neurons and cardiomyocytes [1]. Two patients in previous cohorts died—one during status epilepticus and another of unknown cause, possibly SUDEP; both carried the p.Arg114His variant [2]. This suggests that some *FGF12* pathogenic variants, among other genetic causes, may be a risk factor for early SUDEP [3] (Figure 1B).

*FGF12* gain‐of‐function variants have been associated with epileptic encephalopathy with dysautonomic signs such as apnoea and bradycardia, but the cardiac phenotype of these patients has not been further studied, and ictal asystole was not reported in these patients [3]. The *FGF12* p.(Gly112Ser) variant has only been described in two patients, whereas 19 cases involve the p.(Arg114His) variant. The phenotype associated with p.(Gly112Ser) appears to be milder, with epilepsy onset at 4 months, mild or no developmental delay, and normal brain MRI, consistent with our patient.

Our case highlights the need for cardiac monitoring in DEE patients with *FGF12* variants to detect and manage ictal asystole.

## Conflicts of Interest

The authors declare no conflicts of interest.

## Peer Review

The peer review history for this article is available at https://www.webofscience.com/api/gateway/wos/peer‐review/10.1111/cge.70018.

## Data Availability

The data that support the findings of this study are available on request from the corresponding author. The data are not publicly available due to privacy or ethical restrictions.
